# Crucial factors of the inflammatory microenvironment (IL-1β/TNF-α/TIMP-1) promote the maintenance of the malignant hemopoietic clone of myelofibrosis: an *in vitro* study

**DOI:** 10.18632/oncotarget.9949

**Published:** 2016-06-11

**Authors:** Daria Sollazzo, Dorian Forte, Nicola Polverelli, Marco Romano, Margherita Perricone, Lara Rossi, Emanuela Ottaviani, Simona Luatti, Giovanni Martinelli, Nicola Vianelli, Michele Cavo, Francesca Palandri, Lucia Catani

**Affiliations:** ^1^ Department of Experimental, Diagnostic and Specialty Medicine, Institute of Hematology “L. e A. Seràgnoli”, University of Bologna, Bologna, Italy

**Keywords:** circulating CD34^+^ cells, myelofibrosis, inflammatory microenvironment, migration, survival

## Abstract

Along with molecular abnormalities (mutations in *JAK2*, Calreticulin (*CALR*) and *MPL genes*), chronic inflammation is the major hallmark of Myelofibrosis (MF). Here, we investigated the *in vitro* effects of crucial factors of the inflammatory microenvironment (Interleukin (IL)-1β, Tumor Necrosis Factor (TNF)-α, Tissue Inhibitor of Metalloproteinases (TIMP)-1 and ATP) on the functional behaviour of MF-derived circulating CD34^+^ cells.

We found that, regardless mutation status, IL-1β or TNF-α increases the survival of MF-derived CD34^+^ cells. In addition, along with stimulation of cell cycle progression to the S-phase, IL-1β or TNF-α ± TIMP-1 significantly stimulate(s) the *in vitro* clonogenic ability of CD34^+^ cells from *JAK2^V617^* mutated patients. Whereas in the *JAK2^V617F^* mutated group, the addition of IL-1β or TNF-α + TIMP-1 decreased the erythroid compartment of the CALR mutated patients. Megakaryocyte progenitors were stimulated by IL-1β (*JAK2^V617F^* mutated patients only) and inhibited by TNF-α. IL-1β + TNF-α + C-X-C motif chemokine 12 (CXCL12) ± TIMP-1 highly stimulates the *in vitro* migration of MF-derived CD34+ cells. Interestingly, after migration toward IL-1β + TNF-α + CXCL12 ± TIMP-1, CD34^+^ cells from *JAK2^V617F^* mutated patients show increased clonogenic ability.

Here we demonstrate that the interplay of these inflammatory factors promotes and selects the circulating MF-derived CD34^+^ cells with higher proliferative activity, clonogenic potential and migration ability. Targeting these micro-environmental interactions may be a clinically relevant approach.

## INTRODUCTION

Myelofibrosis (MF) is a life-threatening chronic myeloproliferative neoplasia (MPN) of the hematopoietic stem/progenitor cell (HSPC) clinically characterized by progressive anemia, splenomegaly and constitutional symptoms and by an increased risk to develop acute leukemia (AL). It can arise de novo (primary MF; PMF) or can evolve from Polycythemia Vera (PV; PPV MF) or Essential Thrombocythemia (ET; PET MF) [1–3].

Approximately 50 to 60% of MF patients carry a mutation in the Janus kinase 2 (*JAK2*) gene, while 20–25% of patients show recurrent mutations in the Calreticulin (*CALR*) and an additional 5 to 10% have activating mutations in the myeloproliferative leukemia virus oncogene (*MPL*) gene. Around 10% of patients have non-mutated *JAK2*, *MPL* and *CALR* genes (“triple negative”). Regardless of molecular status, all patients have a deregulation in the JAK/STAT signalling [4–9].

Besides molecular abnormalities, the inflammatory microenvironment has emerged in the last few years as a key-player in MF pathogenesis [10]. Abnormal expression and activity of several cytokines involved in inflammation and immunoregulation are associated with MF [11] and correlate with more severe marrow fibrosis [12, 13], worsening systemic symptoms [14] and decreased survival [15]. Also, the constitutive mobilization of CD34^+^ cells into the peripheral blood has been associated with profound alterations in the CXC chemokine receptor 4 (CXCR4)/C-X-C motif chemokine 12 (CXCL12) axis [16–18]. Up-regulated production of proinflammatory cytokines by HSPCs and surrounding stromal cells generates a microenvironment that selects for the malignant clone [11, 19–23].

Interestingly, HSPCs actively sense pro-inflammatory factors [24]. However, the key players linking inflammation and cancer in MF are still to be defined. Particularly, the plasma levels of Interleukin (IL)-1β, Tumor Necrosis Factor (TNF)-α and Tissue Inhibitor of Metalloproteinases (TIMP)-1 are increased in MF patients [5, 15, 25], but their contribution to disease pathogenesis in MF has been poorly [26] or never investigated. This is also true for the extracellular ATP nucleotide [27]. Under inflammatory conditions, IL-1β stimulates leukocytosis and thrombocytosis by inducing various cytokines (i.e. Granulocyte-Colony Stimulating Factor, IL-6) that are overexpressed in MF; also, IL-1β regulates the survival/proliferation of AL cells [27–30]. IL-1β has been recognized as the main trigger for neural damage and Schwann cell death caused by bone marrow mutant HSPC. Notably, mutant-HSPC-driven niche damage seems to critically contribute to MPN pathogenesis [31]. TNF-α promotes survival of human quiescent bone marrow-derived CD34^+^ Burst Forming Unit-Erythrocyte (BFU-E) and facilitates the clonal expansion of JAK2^V617F^-positive cells in MPNs [26, 32]. TIMP-1, through receptor (CD63) binding, promotes cell survival, differentiation and migration; also, TIMP-1 displays cytokine-like features in the HSPC compartment [33–35]. It was initially found to enhance the proliferation of erythroid cells [36]; also, we recently demonstrated that TIMP-1 increases the clonogenic efficiency of normal CB-derived progenitor cells [37]. Finally, extracellular nucleotides, mainly ATP, are important mediators in inflammation and modulation of cell proliferation, migration and death, including AL CD34^+^ stem/progenitor cells [24, 37–41].

Here, we addressed the functional effects of these pro-inflammatory factors on the *in vitro* behaviour of HSPCs derived from MF patients, with the aim to investigate their putative role in disease pathogenesis.

## RESULTS

### Regardless of mutation status, the plasma levels of IL-1β, TNF-α and TIMP-1 are increased in MF patients

**Figure 1:**
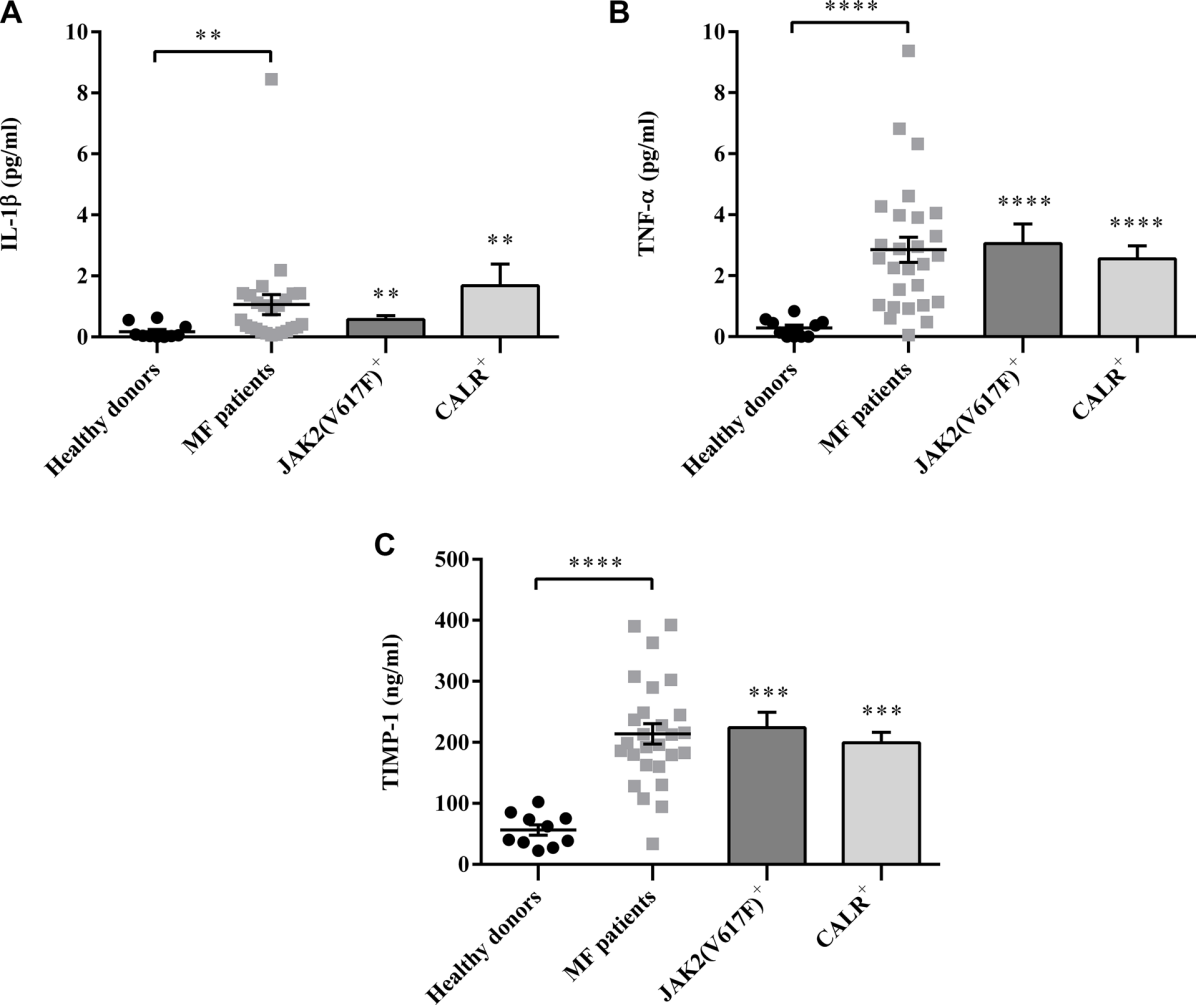
Regardless mutation status, the plasma levels of IL-1β, TNF-α and TIMP-1 are increased in MF patients IL-1β (**A**), TNF-α (**B**) and TIMP-1 (**C**) plasma levels were measured by ELISA in MF patients. (*n* = 26; *JAK2^V617F^* positive *n* =16; *CALR* positive *n* = 10) and healthy controls (*n* = 15). Compared with controls, cytokines plasma levels were significantly increased in MF patients. Of note, there was no significant difference between *JAK2^V617F^* or *CALR* mutated patients. All data are presented as mean ± SEM (**p ≤ 0.01; ***p ≤ 0.001; ****p ≤ 0.0001).

To evaluate the pro-inflammatory profile, selected plasma cytokines were measured. Compared with controls, IL-1β, TNF-α and TIMP-1 plasma levels were significantly increased in MF patients (regardless of IPSS risk stratification values) (Figure 1A, 1B, 1C). We found a trend, albeit not statistically significant (*p = 0.06*), toward increased IL-1β plasma levels in *CALR* mutated patients. Targeting TNF-α and TIMP-1, no significant differences were observed between *JAK2*^V617F^ and *CALR* mutated groups.

### Selected subsets of circulating HSPCs are expanded in MF patients

To determine the extent of the circulating HSPCs compartment according to mutations, we phenotypically analysed the whole blood of MF patients.

**Figure 2:**
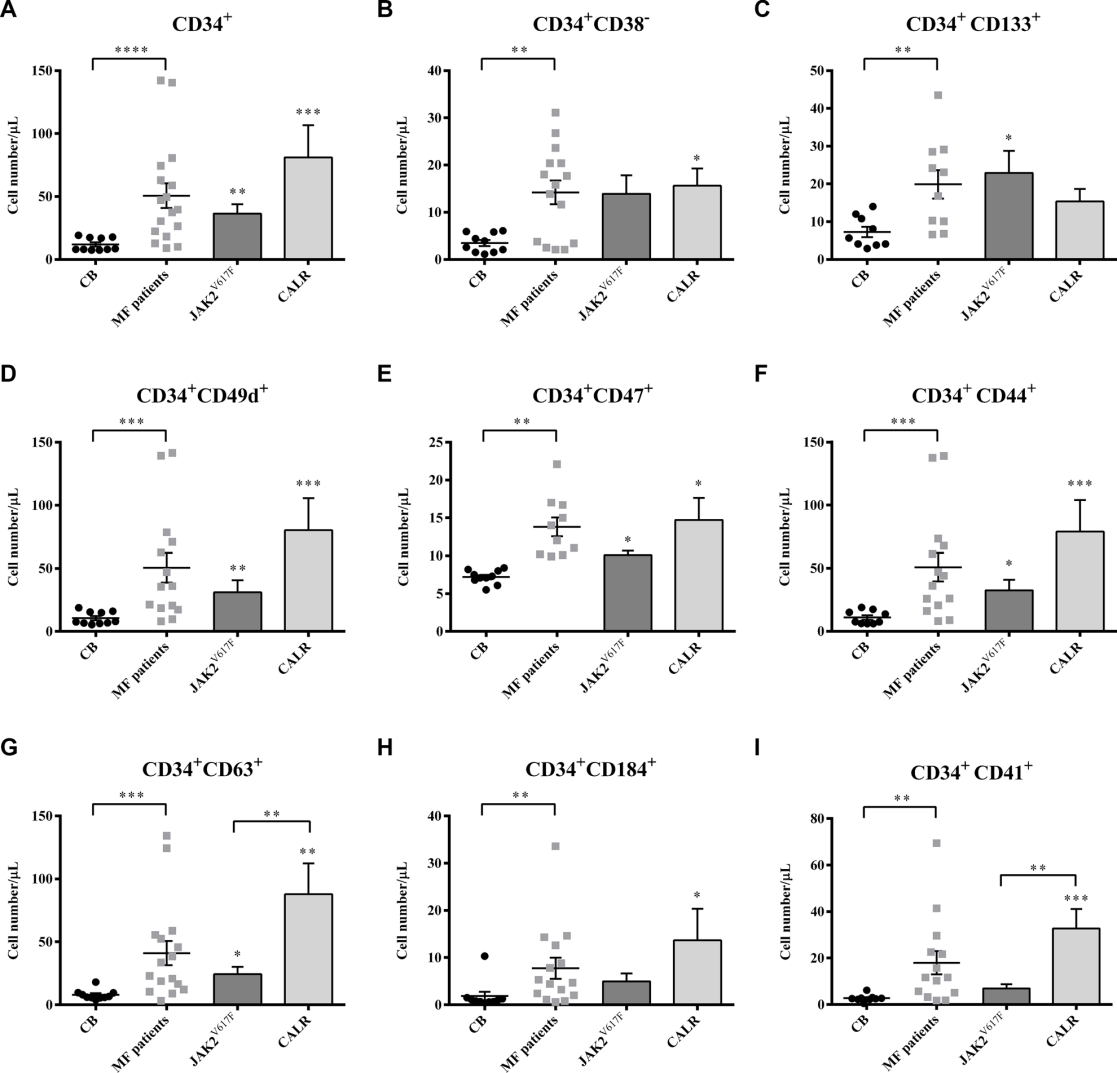
Selected subsets of circulating HSPCs are expanded in MF patients The circulating absolute number of MF (total (*n* = 30) and subdivided into *JAK2^V617F^* (*n* = 20) or *CALR* (*n* = 10) mutated groups) and CB (*n* = 10) CD34^+^ cells coexpressing the CD133, CD49d, CD47, CD44, CD63, CD184 and CD41 antigens together with the CD34^+^ CD38^−^ subset are shown (**A**–**I**). All subsets were increased in MF patients as compared with the CB counterparts. No significant differences were observed between the two mutated groups, except the CD34^+^ CD63^+^ and the CD34^+^ CD41^+^ cells of *CALR* mutated patients which were significantly increased as compared with the *JAK2^V617F^* counterparts. All data are presented as mean ± SEM (*p ≤ 0.05; **p ≤ 0.01; ***p ≤ 0.001; ****p ≤ 0.0001).

Irrespective of mutation status, the mean number of circulating CD34^+^ cells was significantly higher in MF patients than in controls *(p ≤ 0.0001)*. No significant differences were observed between the two mutated groups (Figure 2A). Of note, the number of CD34^+^ cells correlated with IPSS risk in *JAK2*^V617F^ mutated patients (r = 0.88; *p = 0.02*; data not shown).

Along with CD34^+^CD38^−^ and CD34^+^CD133^+^ cells (Figure 2B, 2C), circulating CD34^+^ cells co-expressing adhesion molecules (CD49d, CD47 and CD44; Figure 2D–2F) were also significantly increased in MF patients. Once again, no significant difference was observed between the two mutated groups.

The median number of circulating MF-derived CD34^+^ cells co-expressing the TIMP-1 (CD63) or the CXCL12 receptor (CD184; CXCR4) was significantly higher (*p ≤ 0.001* and *p ≤ 0.01*, respectively) than the CB counterparts (Figure 2G, 2H). *CALR* mutated patients showed increased number of circulating CD34^+^CD63^+^ and CD34^+^CD184^+^ cells compared to *JAK2*^V617F^ mutated patients (*p ≤ 0.01* for CD34^+^CD63^+^) or the CB-counterparts (*p ≤ 0.01* and *p ≤ 0.05,* respectively). CD34^+^CD63^+^ cells of *JAK2*^V617F^mutated patients were also increased compared with the CB-derived cells (*p ≤ 0.05*).

As shown in Figure 2I, circulating megakaryocyte (MK) progenitors (CD34^+^CD41^+^) were also significantly increased (*p ≤ 0.01*). *CALR* mutated patients showed increased number of CD34^+^CD41^+^ cells compared to *JAK2*^V617F^ mutated patients (*p ≤ 0.01* ) or the CB-counterparts (*p ≤ 0.001*).

Of note, except of the decreased expression of CD184 in MF cells, the analysis of mean fluorescence intensity (MFI) of CD133, CD63, CD41, CD49d, CD44 and CD47 antigens on the CD34^+^ cells did not reveal any difference between patients and controls or between the two mutated groups (data not shown).

These data demonstrate that in MF, irrespective of mutation status, there is an *in vivo* expansion of the HSPCs compartment. However, the *CALR* mutated patients show an increased number of circulating CD34^+^CD63^+^ and CD34^+^CD41^+^ cells compared to the *JAK2*^V617F^ mutated counterparts.

### Survival of CD34^+^ cells from MF patients is increased by IL-1β and TNF-α

To investigate whether inflammatory signals may regulate the survival of HSPCs, CD34^+^ cells from MF patients or CB were *in vitro* cultured with the selected pro-inflammatory factors, alone or in combination, at concentrations previously shown to be effective in dose-response experiments (Supplementary Figure 1).

**Figure 3:**
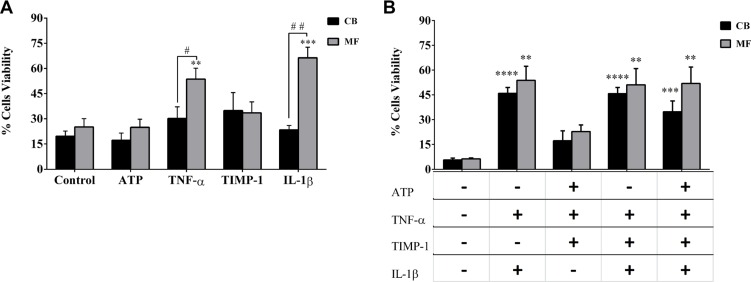
Survival of CD34^+^ cells from MF patients is increased by IL-1β and TNF-α (**A**) CD34^+^ cells from MF patients (*n* = 20) or CB (*n* = 8) were *in vitro* treated for 4 days with factors alone and the percentage of cell viability was assessed after Annexin V/PI staining, as described in Methods. At variance with CB-derived cells, TNF-*α* and IL-1*β* alone significantly stimulated the survival of MF-derived CD34^+^ cells as compared with untreated cells and the CB-derived counterparts. Conversely, ATP and TIMP-1 were ineffective in normal and diseased cells.(**B**) In selected experiments, before Annexin V/PI staining, MF- (*n* = 10) and CB- (*n* = 6) derived CD34^+^ cells were also labeled with a MoAb against the human CD38 antigen and the CD34^+^ CD38^−^ cells were gated and cell viability was analyzed. Once again, multiple combinations of cytokines with IL-1β or TNF-α significantly stimulated the survival of MF- and CB-derived CD34^+^ CD38^−^ cells. Notably, this was not true for ATP+ TNF-α+ TIMP-1. No differences were observed between MF patients and CB. All data are presented as mean ± SEM. (**p ≤ 0.01; ***p ≤ 0.001; ****p ≤ 0.0001 vs untreated cells (CTR)) (^#^*p ≤ 0.05;* ^##^*p ≤ 0.01 vs CB*).

We firstly assessed the effects of factors alone on the *in vitro* survival of CD34^+^ cells. As shown in Figure 3A, the survival of CD34^+^ cells from MF patients was significantly promoted by IL-1β or TNF-α as compared with the CB CD34^+^ cells (*p ≤ 0.01 and p ≤ 0.05*, respectively) or with the untreated MF cells (*p ≤ 0.001* and *p ≤ 0.01,* respectively). No significant differences in survival were observed between the two mutated groups in all tested conditions (data not shown).

As shown in Supplementary Figure 2, the combinations of factors two-by-two significantly promoted the MF-derived CD34^+^ cells survival as compared with untreated cells. However, no significant differences in cell viability were observed as compared with factors alone. Interestingly, the two by two combined factors did not significantly enhance the survival of CB-derived CD34^+^ cells, except for IL-1β + TNF-α (*p ≤ 0.01*). Comparing MF vs CB-derived cells, the survival of MF CD34^+^ cells was significantly enhanced by IL-1β + TIMP-1 (*p ≤ 0.01*) and IL-1β + ATP (*p ≤ 0.01*).

When multiple factors were combined no significant differences were observed between MF and CB-derived CD34^+^ cells. Only TNF-α + TIMP-1 + ATP significantly promoted the survival of *JAK2*^V617F^ CD34^+^ cells as compared with the *CALR* (*p ≤ 0.01*) or CB counterparts (*p ≤ 0.001*) (data not shown).

When we analyzed the CD34^+^ CD38^−^ cells (Figure 3B), we found that multiple factors combinations (particularly those including IL-1β) significantly stimulated the cell survival of MF and CB-derived cells as compared with untreated cells. However, no significant differences were observed between patients/controls (Figure 3B) or the two mutated groups (data not shown)

Taken together these data demonstrate that, regardless of mutation status, the survival of MF-derived CD34^+^ cells is highly stimulated by the *in vitro* treatment with IL-1β or TNF-α. Combinations of pro-inflammatory factors do not have synergistic effects.

### Clonogenic output of circulating MF-derived CD34^+^ cells is positively enhanced by IL-1β + TNF-α ± TIMP-1 combinations

To analyse the functional role of the selected pro-inflammatory cytokines on HSPCs, we investigated their effects on the clonogenic output of circulating MF and CB-derived CD34^+^ cells.

**Figure 4:**
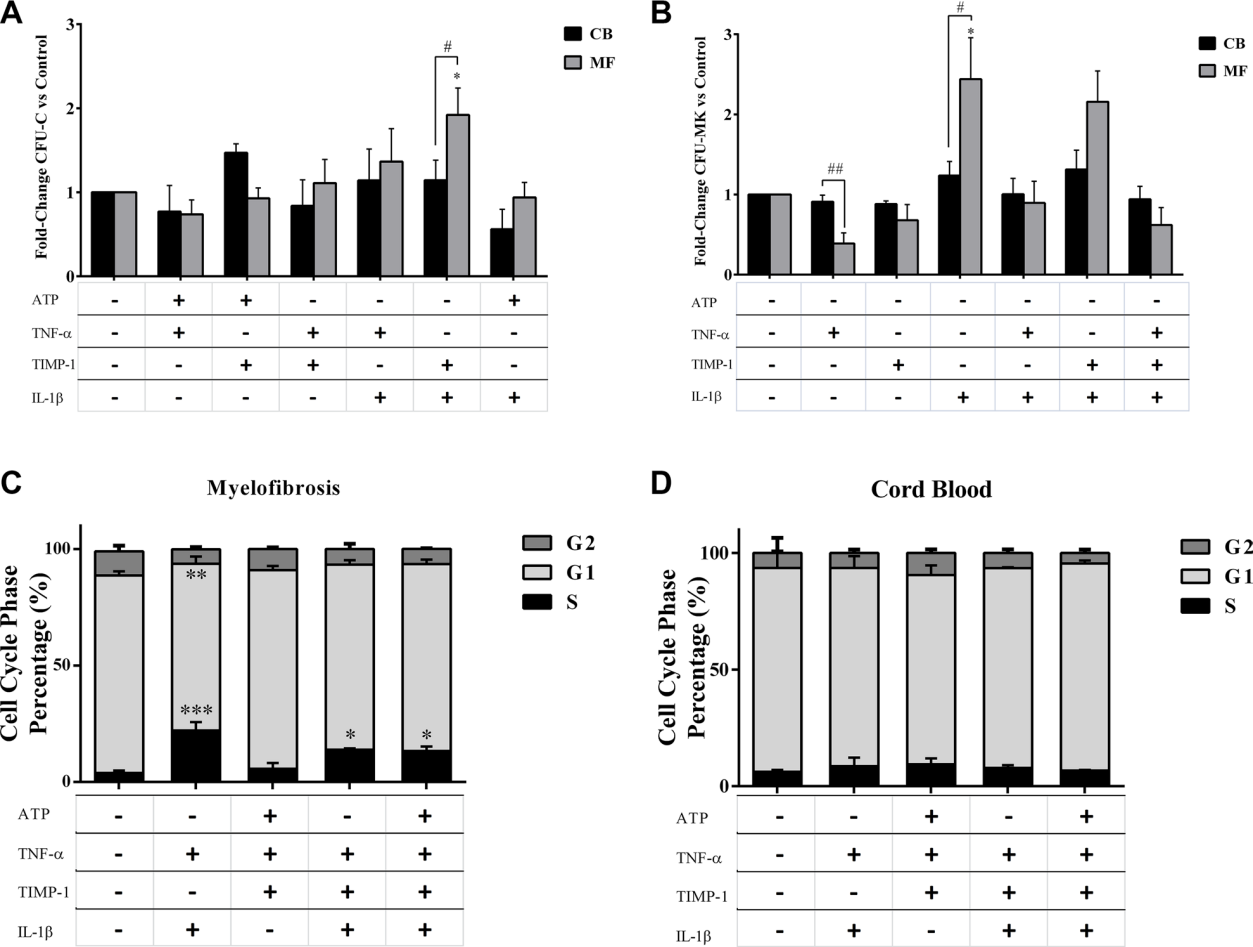
Proliferation of circulating MF-derived CD34^+^ cells is positively enhanced by IL-1β + TNF-α ± TIMP-1 combinations Circulating CD34^+^ cells were isolated from MF patients (*n* = 20) and CB units (*n* = 8) and cultured in the presence of the selected two-by-two pro-inflammatory factors. After 14 days, the total CFU-C output was assessed as described in Methods (**A**). Circulating CD34^+^ cells were isolated from MF patients (*n* = 10) and CB units (*n* = 8) and cultured in the presence or absence of inflammatory factors alone or combined. After 12 days, the CFU-MK growth was assessed as described in Methods (**B**). The results are expressed as growth fold change versus untreated CTR samples. (A) The clonogenic output of the MF-derived CD34^+^ cells was significantly stimulated by the IL-1β + TIMP-1 combination as compared with untreated cells or the CB-derived counterparts. No other combinations of factors two-by-two were effective. The mean number of colonies in MF-derived and CB-derived untreated samples was 59 ± 8 and 63 ± 6, respectively. (B) The MF-derived CFU-MK growth was significantly inhibited by TNF-α. By contrast, IL-1β has stimulatory activity on MK colony formation. Factors in combination did not significantly modify the growth of patients/CB CFU-MK as compared with factors alone. Factors alone or in combination did not significantly modify the CFU-MK growth of the CB counterparts. The mean number of CFU-MK in MF- and CB-derived untreated samples was 26 ± 11 and 46 ± 10, respectively. In (**C** and **D** ) are shown the results of cell-cycle analysis of MF-derived (*n* = 10) and CB-derived (*n* = 8) CD34^+^ cells after *in vitro* incubation for 24 hours in the presence or absence of various combinations of pro-inflammatory factors. Results are expressed as the percentage of cells in different phases of the cell cycle. IL-1β plus TNF-α highly promote cell cycling of CD34^+^ cells from MF patients. IL-1β + TNF-α + TIMP-1 and IL-1β + TNF-α + TIMP-1 + ATP were also effective (C). Conversely, no significant differences were observed when CB-derived cells were analysed (D). All data are presented as mean ± SEM. (*p ≤ 0.05; **p ≤ 0.01; ***p ≤ 0.001 vs untreated cells) (^#^*p ≤ 0.05; ^##^p ≤ 0.01 vs CB*).

Factors alone did not induce a significant CFU-C growth from MF CD34^+^ cells (data not shown). However, when MF-derived CD34^+^ cells were tested in the presence of combinations of factors two-by-two, the IL-1β + TIMP-1 combination was the only one effective in stimulating the CFU-C growth as compared with untreated cells (*p ≤ 0.05*) or CB-derived CD34^+^ cells (*p ≤ 0.05*) (Figure 4A). IL-1β + TNF-α and IL-1β + TIMP-1 significantly promoted the BFU-E growth of the MF-derived CD34^+^ cells as compared with the untreated samples and the CB counterparts. The CFU-GM growth was positively enhanced by IL-1β + TIMP- 1 (Supplementary Figure 3A, 3B). Of note, when combinations of multiple factors were tested, only IL-1β + TNF-α + TIMP-1 significantly promoted the CFU-C growth (*p ≤ 0.05)* of CD34^+^ cells from CB (data not shown).

As shown in Figure 4B, when the growth of MK progenitors was investigated in the presence of inflammatory factors alone, we found that, at variance with CB, the MF-derived CFU-MK growth was significantly inhibited by TNF-α. By contrast, IL-1β has stimulatory activity on MK colony formation. Factors in combination did not significantly modify the growth of patients/CB pure CFU-MK as compared with factors alone.

We also examined the cell-cycle profile of MF-derived and CB-derived CD34^+^ cells after *in vitro* exposure to the cytokines. We found that most of the untreated CD34^+^ cells from MF patients were in a dormant state. Factors alone did not significantly increased the percentage of CD34^+^ cells in S phase as compared with untreated cells, both in MF patients and CB (data not shown). Conversely, in MF patients, irrespective of mutation status, cell-cycle progression was observed in presence of various cytokines combinations, with the notable exception of ATP + TNF-α + TIMP-1 (Figure 4C, 4D).

### Opposite effects of pro-inflammatory cytokines on clonogenic potential of CD34^+^ cells from *JAK2^V617F^* or *CALR* mutated patients

When clonogenic potential was analysed according to mutation status and in the presence of pro-inflammatory factors alone, no differences were observed between the two mutated groups. Colony composition analysis demonstrated that only IL-1β enhanced the erythroid compartment of the *JAK2*^V617F^ mutated group (Supplementary Figure 4A and 4B).

**Figure 5:**
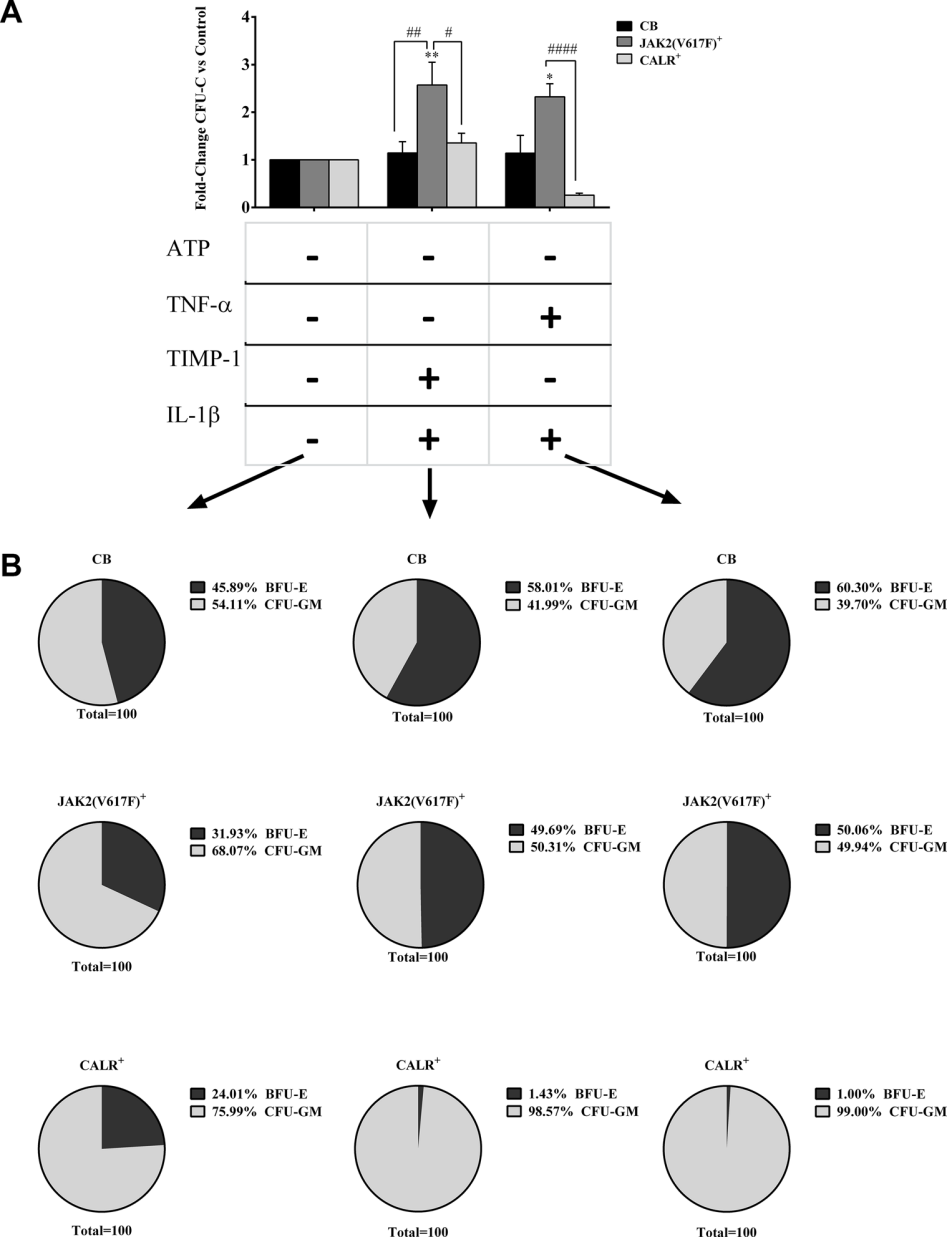
Opposite effects of pro-inflammatory cytokines on cells from *JAK2^V617F^* or *CALR* mutated patients (**A**) When clonogenic activity was analyzed according to mutation status, the CFU-C growth of *JAK2^V617F^* mutated patients was significantly up-regulated by IL-1β + TIMP-1 and IL-1β + TNF-α as compared with untreated control samples, the *CALR* mutated counterparts and the CB-derived cells (only IL-1β + TIMP-1). The results are expressed as growth fold change versus untreated CTR samples. All data are presented as mean ± SEM. (*p ≤ 0.05; **p ≤ 0.01 vs untreated cells) (^#^*p ≤ 0.05;* ^##^*p ≤ 0.01;* ^####^*p ≤ 0.0001 vs CB-derived cells*) (**B**) When colony composition was analyzed according to mutation status, we found that the erythroid compartment of the untreated samples was reduced in both mutated groups as compared with the CB counterparts. However, no significant differences were observed between the two mutated groups. Interestingly, in the *JAK2^V617F^* mutated group, the addition of IL-1β + TIMP-1 and IL-1β + TNF-α enhanced the erythroid compartment as compared with untreated samples. Conversely, some cytokines combinations significantly impaired BFU-E growth in *CALR* mutated patients. The results are expressed as mean percentage of CFU-GM/BFU-E as compared with the total CFU-C count.

By contrast, (Figure 5A), the combination of IL-1β + TIMP-1 and IL-1β + TNF-α significantly promoted the CFU-C growth of *JAK2*^V617F^ mutated patients compared with the *CALR* mutated counterparts. Similar results were obtained when CFU-GM and BFU-E growth were distinctly analysed (data not shown).

When colony composition was analysed according to mutation status (Figure 5B), both mutated groups showed reduced BFU-E growth of the untreated samples as compared with the CB counterparts. Interestingly, in the *JAK2*^V617F^ mutated group, the addition of different combinations of pro-inflammatory factors enhanced the erythroid compartment as compared with untreated samples. Conversely, some cytokines combinations significantly decreased BFU-E growth in *CALR*-mutated patients.

Multiple combinations did not significantly modified the clonogenic activity and colony composition of the two mutated groups (data not shown).

When we analysed the percentage of *JAK2*^V617F^ and *CALR* mutant colonies in the absence or presence of IL-1β + TNFα, we found that the percentage of *JAK2*^V617F^, but not *CALR*, mutated colonies was increased (data not shown).

MK progenitors of *JAK2*^V617F^ mutated patients were highly stimulated by IL-1β alone. By contrast, TNF-α significantly inhibited the CFU-MK growth of both *JAK2*^V617F^ and *CALR* mutated patients as compared with CB counterparts (Supplementary Figure 5).

Taken together these results demonstrate that the hemopoietic function of MF-derived CD34^+^ cells is highly promoted by the IL-1β or TNF-α ± TIMP-1 combinations, even though TNF-α alone show inhibitory effects on MK progenitors. Interestingly, whereas in the *JAK2^V617F^* mutated group, the addition of various combinations of growth factors decreased the erythroid compartment of the *CALR* mutated patients.

### IL-1β and TNF-α significantly promote migration of MF-derived CD34^+^ cells showing enhanced clonogenic ability after migration in *JAK2^V617F^* mutated patients

**Figure 6:**
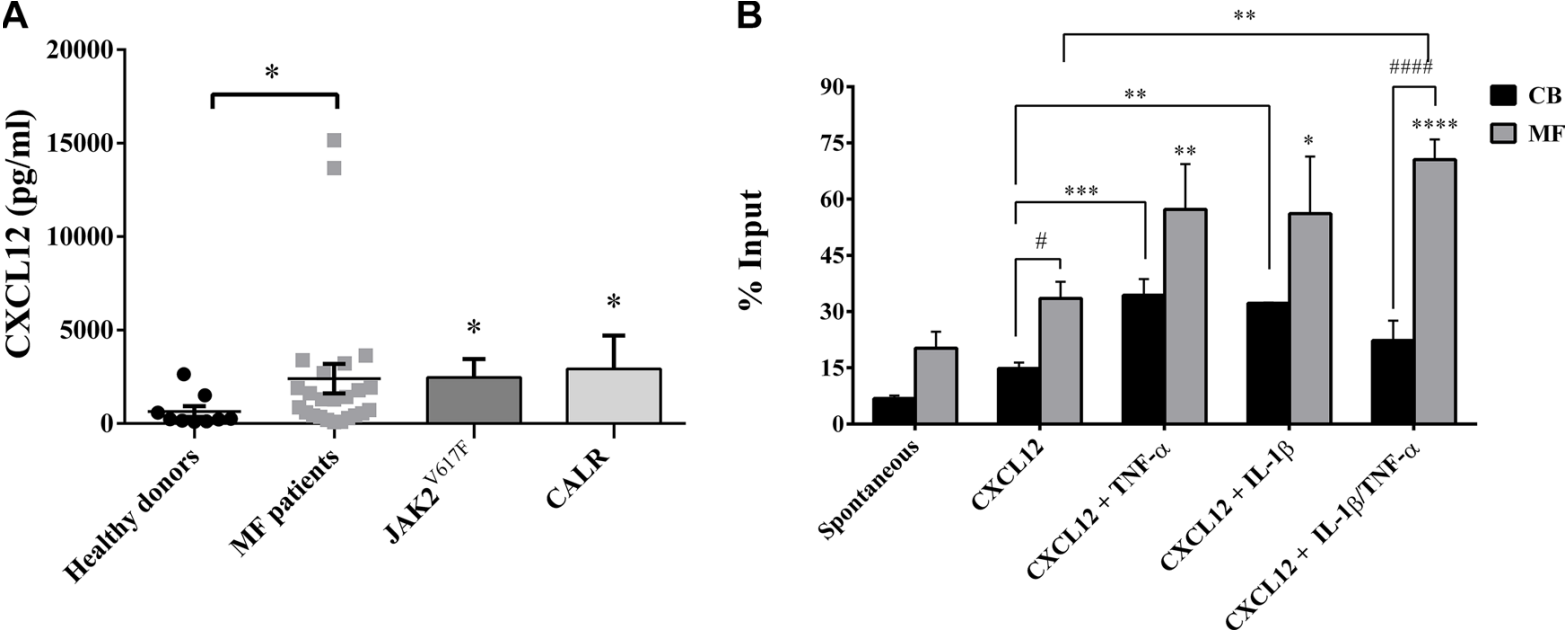
IL-1β and TNF-α significantly increases migration of MF-derived CD34^+^ cells (**A**) CXCL12 plasma levels of MF patients (total *n* =24; *JAK2^V617F^* mutated patients *n* = 16; *CALR* mutated patients *n* = 8) and controls (*n* = 10). Regardless mutation status, CXCL12 concentration was significantly higher in MF patients (*p ≤ 0.05 vs controls). (**B**) When cells were migrated toward CXCL12 alone, an increased migration ability was observed in MF-derived (*n* = 15) CD34^+^ cells as compared with the CB-derived (*n* = 8) counterparts. The addition of inflammatory factors alone (IL-1β/TNF-α) plus CXCL12 significantly increased the migratory behaviour of MF-derived CD34^+^ cells as compared with CXCL12 alone. IL-1β + TNF-α synergistically enhanced the migratory behaviour of CD34^+^ cells as compared with spontaneous migration (****p < 0.0001), CXCL12 alone (**p <0.001) and the CB-counterpart (^####^*p <0.0001).* Results are expressed as mean percentages ± SEM of input. (**p ≤ 0.01; ***p ≤ 0.001 vs CXCL12 alone for CB-derived CD34^+^ cells) (*p ≤ 0.05; **p ≤ 0.01; ****p < 0.0001 vs spontaneous migration for MF-derived CD34^+^ cells) (^#^*p ≤ 0.05; ^####^p <0.0001 vs CB*).

To evaluate whether selected pro-inflammatory factors may differentially regulate the migratory ability of HSPCs from MF patients, we firstly analyzed the plasma concentration of CXCL12. CXCL12 plasma level was markedly higher in patients than in controls, either total (*p ≤ 0.05*) or subdivided according to mutation status (*JAK2*^V617F^ *p ≤ 0.05; CALR p ≤ 0.05*). Conversely, no significant differences were observed between the two mutated groups (Figure 6A).

To mirror the *in vivo* pattern of MF, we set up *in vitro* migration experiments in the presence of the identified inflammatory factors and CXCL12. The migration rate of MF- or CB-derived CD34^+^ cells toward inflammatory factors alone (without CXCL12) was not significantly different from that of untreated cells (data not shown).

As shown in Figure 6B, CXCL12 significantly increased the migratory behaviour of MF-derived CD34^+^ cells as compared with CB counterparts (*p ≤ 0.05*). The addition of IL-1β or TNF-α + CXCL12 shows a trend toward increased migration of CD34^+^ cells from MF patients, but doesn't reach statistical significance. At variance with CB derived cells, the addition of both cytokines significantly increased the migratory potential of CD34^+^ cells from MF patients (*p ≤ 0.01*). No differences were observed between the two mutated groups (data not shown).

The addition of TIMP-1 and ATP alone or inflammatory factors two by two in the presence of CXCL12 did not significantly increase the migration ability of MF-derived CD34^+^ cells as compared with CXCL12 alone (data not shown). The migratory behavior of the MF-derived CD34^+^ cells toward multiple combinations of factors was significantly enhanced as compared with the CB-derived counterparts in all tested combinations. Conversely, CB-derived CD34^+^ cells were almost insensitive (Supplementary Figure 6).

**Figure 7:**
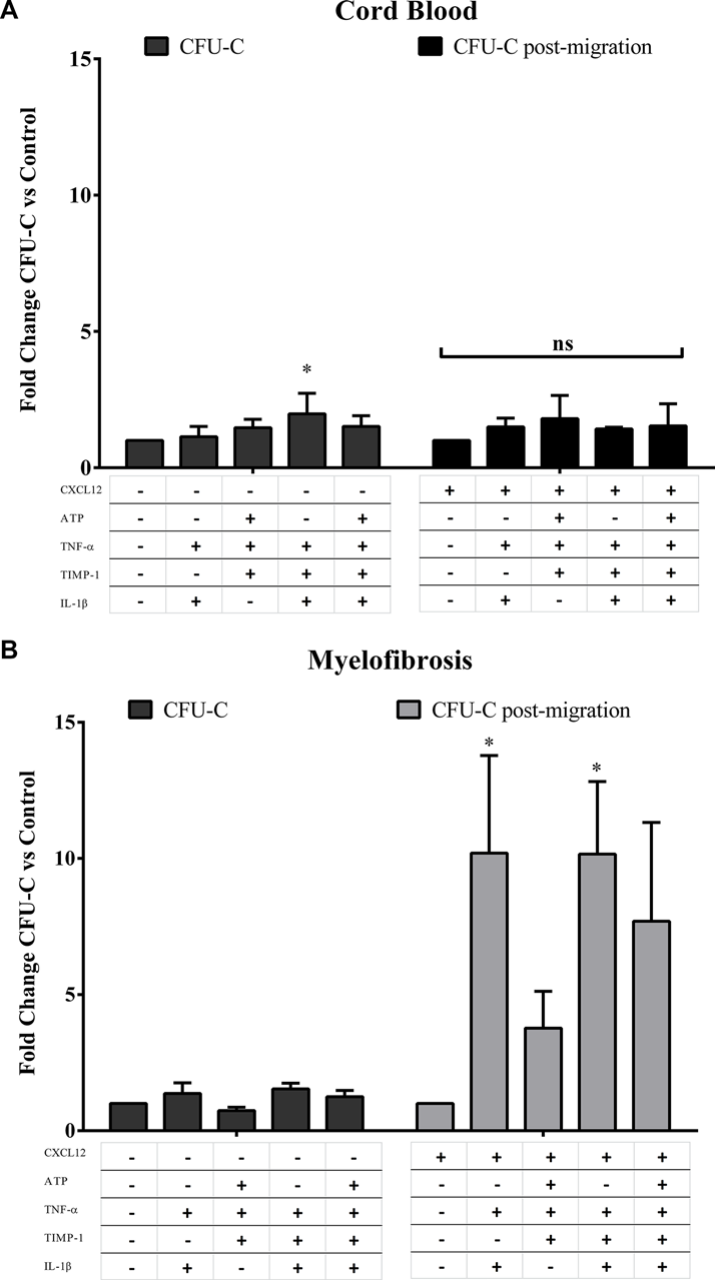
The clonogenic output of MF-derived CD34^+^ cells after migration toward IL-1β + TNF-α + CXCL12 ± TIMP-1 is potently enhanced Panels (**A** and **B**) show the clonogenic potential of CB-derived (A; *n* = 6) and MF-derived CD34^+^ cells (B; *n* = 14) at baseline with or without various combinations of pro-inflammatory factors (CFU-C) and after migration toward CXCL12 alone or various combinations of pro-inflammatory factors + CXCL12 (CFU-C post-migration). After migration toward IL-1β + TNF-α + CXCL12 ± TIMP-1, the MF-derived, but not CB-derived, CD34^+^ cells show significantly increased clonogenic potential. Results are expressed as mean fold change of CFU-C ± SEM. (*p ≤ 0.05 vs untreated cells (A) and CXCL12 alone (B)).

To evaluate whether migrated cells toward various pro-inflammatory gradients show different hemopoietic function, we also tested the clonogenic potential of CD34^+^ cells from MF patients or CB after migration toward CXCL12 alone or CXCL12 plus various combinations of factors (Figure 7A, 7B). Interestingly, at variance with the CFU-C growth of unmigrated HSPCs from MF patients, IL-1β + TNF-α + CXCL12 and IL-1β + TNF-α + TIMP-1 + CXCL12 selected a subset of MF-derived CD34^+^ cells with higher clonogenic potential as compared with CXCL12 alone (*p ≤ 0.05*, respectively) (Figure 7B). Conversely, the clonogenic output of CB-derived CD34^+^ cells after migration toward various combinations of pro-inflammatory factors was unaffected (Figure 7A). Notably, according to mutation status, the CFU-C post migration assay demonstrated once again that various combinations of pro-inflammatory factors significantly stimulate the clonogenic ability of migrated CD34^+^ cells from *JAK2*^V617F^, but not *CALR,* mutated patients (Supplementary Figure 7).

When the number of granulocyte and erythroid colonies were analysed individually, only BFU-E growth was significantly increased with respect to controls (*p ≤ 0.05*) after cells were migrated toward IL-1β + TNF-α + CXCL12 ± TIMP-1. Of note, IL-1β + TNF-α + TIMP-1 + CXCL12 significantly stimulated also CFU-GM growth as compared with CXCL12 alone (*p ≤ 0.05*) (data not shown).

Taken together these results demonstrate that, irrespective of mutation status, IL-1β + TNF-α + CXCL12 ± TIMP-1 selectively enhance the migratory ability of MF-derived CD34^+^ cells. Interestingly, IL-1β + TNF-α + CXCL12 ± TIMP-1 promotes and selects the circulating HSPCs of *JAK2*^V617F^ mutated patients with higher clonogenic potential.

## DISCUSSION

Here, we evaluated the *in vitro* effects of four main crucial factors of the inflammatory microenvironment (IL-1β, TNF-α, TIMP-1 and ATP) on survival, clonogenic output and migration ability of MF HSPCs.

First, this study demonstrates that, regardless of mutation status, IL-1β, TNF-α and TIMP-1 are increased in the plasma of MF patients and the presence of IL-1β, TNF-α ± TIMP-1 confers a survival advantage of MF-derived HSPCs. Second, HSPCs from *JAK2*^V617F^ show *in vitro* enhanced proliferation over untreated cells and the CB counterparts in response to IL-1β, TNF-α ± TIMP-1 exposure (alone or, mostly, in combination). Accordingly, IL-1β + TNF-α stimulates cell cycle progression of MF-derived CD34^+^ cells to the S-phase. Third, IL-1β + TNF-α combination promotes the *in vitro* migration of MF-derived HSPCs. Interestingly, after migration toward IL-1β + TNF-α + CXCL12 ± TIMP-1, MF-derived CD34^+^ cells show increased clonogenic ability as compared with CXCL12 alone or the CB counterparts. This finding was mainly due to stimulation of the clonogenic growth of HSPCs from *JAK2*^V617F^ mutated patients.

TNF-α has already been shown to facilitate clonal expansion of *JAK2*^V617F^-positive cells in MF [26]. The results of this study provide new evidences that, in addition to TNF-α, IL-1β and TIMP-1 promote the *in vitro* maintenance of the HSPCs.

Mutation status is associated with dysregulated hemopoietic function (clonogenic output and colony composition) of MF-derived CD34^+^ cells in presence of IL-1β + TNF-α ± TIMP-1. Specifically, when pro-inflammatory factors were added in culture, CD34^+^ cells from *JAK2^V617F^* mutated patients showed increased clonogenic potential and increased size of the erythroid progenitors compartment as compared with the *CALR*-mutated counterparts. Along with the CFU-C growth, IL-1β stimulates the *in vitro* growth of MK progenitors of *JAK2^V617F^* mutated patients only. Consistent data on single cell assays suggested that HSC self-renewal capacity is negatively affected by *JAK2^V617F^*, but progenitor cells have increased proliferation capacity [42]. In addition, these findings can be related to the fact that *JAK2*^V617F^ mutant, in contrast with the *CALR* counterpart, activates not only the MK cell line but also the erythroid and granulocytic lineages [43].

Despite increased frequency of MK progenitors (CD34^+^CD41^+^ cells) in the PB of *CALR* mutated patients, we clearly demonstrate that the hemopoietic function of CD34^+^ cells from *CALR* mutated patients is unmodified (megakaryocytic compartment) or significantly inhibited (erythroid compartment) by IL-1β or various combinations of inflammatory factors including IL-1β. It is therefore likely that these functional abnormalities may contribute to explain the lower haemoglobin concentration that is displayed by *CALR*-positive patients compared to *JAK2^V617F^*-mutated patients [8]. Interestingly, at variance with *JAK2*^V617F^, *CALR* mutants moderately activate the PI3K/AKT pathway, a critical determinant of erythropoiesis and megakaryocytopoiesis [43–46].

Of note, we also found increased number of circulating CD34^+^CD63^+^ cells in MF. However, despite TIMP-1 alone was ineffective, various combinations including TIMP-1 increased the proliferation/migration of MF-derived CD34^+^ cells. This finding was not due to upregulated expression of CD63 receptor on MF-derived CD34^+^ cells after exposure to IL-1β and/or TNF-α (data not shown). It is therefore likely that downstream intracellular signaling pathways are hyperactivated and stimulate clonogenic activity.

Overall, our data indicate that the *in vitro* behavior of the MF-derived HSPCs can be upregulated by regulatory signals provided by the microenvironment and, specifically, through the cooperation between various pro-inflammatory factors. Therefore, the increased number of HSPCs in the peripheral blood of MF may be due not only to the displacement of HSPCs from bone marrow into peripheral blood, but also to the proliferative/survival signals coming from the pro-inflammatory factors within the peripheral blood niche. As a consequence, the pro-inflammatory microenvironment emerges as central site for cell division and proliferation.

In conclusion, the *in vitro* interplay between identified pro-inflammatory cytokines, which are abnormally increased, promotes and selects the circulating MF-derived HSPCs with higher proliferative activity, clonogenic potential and migration ability. Thus, it is likely that the *in vivo* inflammatory niche plays a key role in the maintenance of the malignant hemopoietic clone. Targeting these inflammatory micro-environmental interactions may be a clinically relevant approach for MF.

## MATERIALS AND METHODS

### Patients and samples

Peripheral blood (PB) was obtained from 10 normal age-matched volunteers and 36 patients with MF in chronic phase. Patients characteristics according to mutational status are shown in Table 1. At the time of the study, patients were at diagnosis (19 cases) or untreated for at least two months. Previous therapies were: hydroxyurea (12 cases) and Ruxolitinib (3 cases). The diagnosis of MF was made according to WHO 2008 criteria [47]. Patients and controls provided written informed consent for the study. This study was approved by the medical Ethical Committee of the University Hospital of Bologna and was conducted in accordance with the Declaration of Helsinki. During the last trimester of pregnancy, an increased number of CD34^+^ HSPC are mobilized from the fetal liver and can be found in the circulating blood, including umbilical cord blood (CB). Therefore, since HSPC trafficking characterizes both the PB of MF patients and CB, we choose this physiological source for comparison. CB collections (14 cases) from normal full-term deliveries were provided by the Cord Blood Bank of the University Hospital of Bologna after written informed consent.

**Table 1:** Patients characteristics according to mutational status

Characteristics	*JAK2^V617F^*-mutated patients (no. 23)	*CALR* mutated patients (no.13)	*P* value
Median age, years (range)	65 (40–82)	73 (70–84)	**0.01**
Male sex, n° (%)	12 (52%)	7 (54%)	1
Median allele burden, % (range)	89 (0,4–99)	56,5 (47–98)	0.07
Median WBC,×10^9^/L (range)	8,6 (2,5–157,6)	6,6 (2,3–16)	0.48
Median hemoglobin, g/dL (range)	11,9 (8,6–15,1)	9,3 (7,7–12,7)	**0.04**
Median platelet count, ×10^9^/l (range)	270 (41–707)	198 (86–419)	0.44
High/intermediate 2 IPSS category, n° (%)	12 (52)	7 (54)	1
Unfavorable karyotype, n° (%)	9 (39)	3 (23)	0.46
PMF diagnosis, n° (%)	14 (61)	9 (69)	0.7
BM fibrosis grade ≥ 2, n° (%)	14 (61)	13 (100)	**0.03**
Patients with splenomegaly, n° (%)	19 (83)	10 (77)	0.7
Median follow-up, months (range)	45 (1,9–114,9)	48 (10–136,3)	0.24

### Cell isolation

PB, anticoagulated with ethylenediamine tetraacetic acid (EDTA), was obtained from patients/controls. Mononuclear cells (MNCs) were separated from MF and CB samples by stratification on Lympholyte-H 1.077 g/cm^3^ gradient (Gibco-Invitrogen, Milan, Italy), followed by red blood cell lysis for 15 min at 4°C. MNCs were then processed on magnetic columns for CD34^+^ cell isolation (mean purity 94% ± 5%) (MACS CD34 Isolation kit; Miltenyi Biotech, Bologna, Italy), as previously described [37].

### Plasma levels measurement of selected circulating cytokines

We measured the cytokines plasma levels of patients/controls by ELISA, according to the manufacturer's instructions. EDTA-anticoagulated PB was centrifuged for 15 minutes at 1000 g within 30 minutes of collection. The plasma was then collected and stored at −80°C until quantification. In particular, the TIMP-1 ELISA kit was provided from Boster Immunoleader (Boster Biological Technology Co., Pleasanton, CA, USA) and CXCL12 ELISA kit from Krishgen ByoSistems (Ashley CT, Whittier, CA, USA). The Ciraplex^TM^ immunoassay kit/Human 9-Plex Array (Aushon BioSystems, Billerica, MA, USA) was used for the measurement of various cytokines including IL-1β and TNF-α.

### Phenotype of circulating CD34^+^ cells

The phenotype of circulating CD34^+^ cells was evaluated in PB from MF patients and in CB samples by conventional immunofluorescence, as previously described (48). Antibodies used to characterize the CD34^+^ cells are listed in Supplementary Table 1. A minimum of 1 × 10^4^ CD34^+^ cells were acquired by flow cytometer BD Accuri C6 (Becton Dickinson). Analysis was performed excluding cellular debris in a SSC/FSC dot plot. The percentage of positive cells was calculated subtracting the value of the appropriate isotype controls. The absolute number of positive cells/mL was calculated as follows: percentage of positive cells × White Blood Cells count/100.

### Apoptosis assay

Freshly isolated CD34^+^ HSPCs (2–5 × 10^5^) from MF patients or CB units were maintained in RPMI 1640 with 10% FBS, with or without IL-1β (1,10 ng/mL), TNF-α (10,100 ng/mL), TIMP-1 (100,300 ng/mL), and ATP (100,1000 μM), alone or in different combinations. After 4 days, cells were stained for 15 min at RT with Annexin-V-FLUOS Staining Kit (Roche, Penzberg, Germany). Samples were then immediately analyzed by BD Accuri C6 (BD Bioscience). Results are expressed as percentage of live cells compared to the whole cells.

### Erythroid and granulocytic progenitors assays

MF/CB-derived CD34^+^ cells were cultured *in vitro* to achieve hematopoietic cell differentiation and the formation of multi-lineage colony-forming units (CFU-Cs), including colony forming unit-granulocyte macrophage (CFU-GM) and BFU-E. Specifically, CD34^+^ cells were seeded in methylcellulose-based medium (human StemMACS HSC-CFU lite w/Epo, Miltenyi Biotech) at 5 × 10^2^ cells/mL in 35-mm Petri dishes in the presence or absence of the selected pro-inflammatory factors: TIMP-1 (100 ng/mL; Thermo Scientific, Pierce Biotechnology, Rockford, IL, USA), ATP (1000 μM; Sigma Aldrich, Milan, Italy), TNF-α (10 ng/mL; Thermo Scientific) and IL-1β (1 ng/mL; Thermo Scientific), alone or in combination. After 2 weeks of incubation at 37°C in 5% humidified CO_2_ atmosphere, CFU-C growth was evaluated by standard morphologic criteria using an inverted microscope (Axiovert 40, Zeiss).

### Megakaryocytic progenitors assay

Megakaryocytic colonies (Colony Forming Unit-Megakaryocyte (CFU-MK)) were obtained using MegaCult™-C assay (Stem Cell Technologies; Vancouver, BC, Canada), according to the manufacturer's protocols. Briefly, 5 × 10^3^ MF/CB-derived CD34^+^ cells were seeded in a collagen-based medium in double chamber slides in the presence or absence of the inflammatory factors, alone or in combination. Cultures were incubated for 12 days and then dehydrated, fixed and stained with a primary antibody to the MK-specific antigen GPllb/llla (CD41) linked to a secondary biotinylated antibody-alkaline phosphatase avidin conjugated detection system. CFU-MK were counted using a light microscope.

### Cell cycle analysis

A total of 10^6^ CD34^+^ cells was maintained in Roswell Park Memorial Institute (RPMI)-1640 (Lonza) supplemented with 10% fetal bovine serum (FBS Thermo Fisher Scientific, Waltham, MA USA). Cells were resuspended in complete medium at a concentration of 1 × 10^6^/mL, and primed for 24 hours with the pro-inflammatory cytokines (1 ng/mL IL-1β, 10 ng/mL TNF-α, 100 ng/mL TIMP-1, 1000 μM ATP), alone or in combination. Treated cells were first permeabilized with NP-40 (15 min at RT) and then labeled with propidium iodide (PI)/RNAse staining kit (BD Bioscience) for 15 min at RT, in the dark. The DNA content was assessed by BD Accuri C6 (BD Bioscience) and results were analyzed by FCS express 4 software.

Changes in the cell-cycle distribution were evaluated using PI. The percentage of cells in the G0/G1, S, and G2/M phases was determined by measuring simultaneously the DNA and RNA total cellular content.

### Migration assay

Migration of MF/CB purified CD34^+^ cells was assayed towards a CXCL12 gradient (150 ng/mL) in transwell chambers (diameter 6.5 mm, pore size 8 μm; Costar; Corning), as previously described [38]. Briefly, 50 μl of RPMI 1640 plus 10% FBS containing 0,5 × 10^5^ cells were added to the upper chamber and 150 μl of medium with or without CXCL12 ± IL-1β (1 ng/mL), TNF-α (10 ng/mL), TIMP-1 (100 ng/mL), and ATP (1000 μM) (alone or in combination) were added to the bottom chamber. After overnight incubation at 37°C in 5% humidified CO2 atmosphere, inserts (upper chambers) were removed and cells transmigrated into lower chamber were recovered and counted by Trypan Blue exclusion test in a Neubauer chamber using an inverted microscope (Nikon) with a 10×magnification. The amount of migrated cells was expressed as a percentage of the input, applying the following formula: (number of migrated cells recovered from the lower compartment/total number of cells loaded in the upper compartment) × 100. In addition, migrated cells were assayed in methylcellulose-based medium for their ability to form hematopoietic colonies (as above described).

### Mutation analysis

*JAK2*^V617F^ allele-burden was assessed in granulocyte DNA by quantitative polymerase chain reaction–based allelic discrimination assay (ipsogen *JAK2* MutaQuant Kit) on 7900 HT Fast Real Time PCR System (Applied Biosystem) [49]. *CALR* exon 9 sequencing was performed by Next Generation Sequencing (NGS) approach with GS Junior (Roche-454 platform); analysis was carried out with AVA Software (GRCh38 as reference) [50]. Rare *CALR* mutations identified by NGS were confirmed by Sanger sequencing.

Individual colonies were harvested at day 12–14 from 3 *JAK2*^V617F^ and 3 *CALR* mutated patients (20 individual colonies each condition). Molecular characterization of single colonies was performed on DNA extracted using REPLI-g Single Cell Kit (QIAGEN, Marseille, France), which provides accurate genome amplification from single cells or limited samples with high efficiency. Briefly, 4 μL of cell material (supplied with PBS) were firstly lysed. After denaturation, isothermal amplification reaction was performed and amplified DNA was used for *JAK2^V617F^* and *CALR* mutations assessment, as above described.

### Cytogenetic analysis

Chromosome banding analysis was performed on bone marrow cells by standard banding techniques according to the International System for Human Cytogenetic Nomenclature. At least 20 metaphases were required. Unfavourable karyotype included complex karyotype or single or two abnormalities including +8, −7/7q-, i(17q), −5%5q-, 12p-, inv(3) or 11q23 rearrangement [51, 52].

### Statistical analysis

Numerical variables have been summarized by their median and range, and categorical variables by count and relative frequency (%) of each category. Comparisons of quantitative variables between groups of patients were carried out by the nonparametric Wilcoxon rank-sum test. All *p* values were considered statistically significant when ≤ 0.05 (2-tailed). Statistical analyses were performed using Graphpad (Graphpad Software Inc., La Jolla, CA) and SPSS software (PASW Statistics for Windows, Version 18.0. Chicago, IL).

## SUPPLEMENTARY MATERIAL FIGURES AND TABLE


