# Supplementary material for: Crucial factors of the inflammatory microenvironment (IL-1β/TNF-α/TIMP-1) promote the maintenance of the malignant hemopoietic clone of myelofibrosis: an in vitro study
Source: Oncotarget. 2016 Jun 11;7(28):43974–88. doi: 10.18632/oncotarget.9949 (PMC5190072; doi:10.18632/oncotarget.9949)
Supplement: Supplementary file 1 [file oncotarget-07-43974-s001.pdf]

Crucial factors of the inflammatory microenvironment (IL-1β/TNF-α/TIMP-1) promote the maintenance of the malignant hemopoietic clone of myelofibrosis: an *in vitro* study

Supplementary Materials

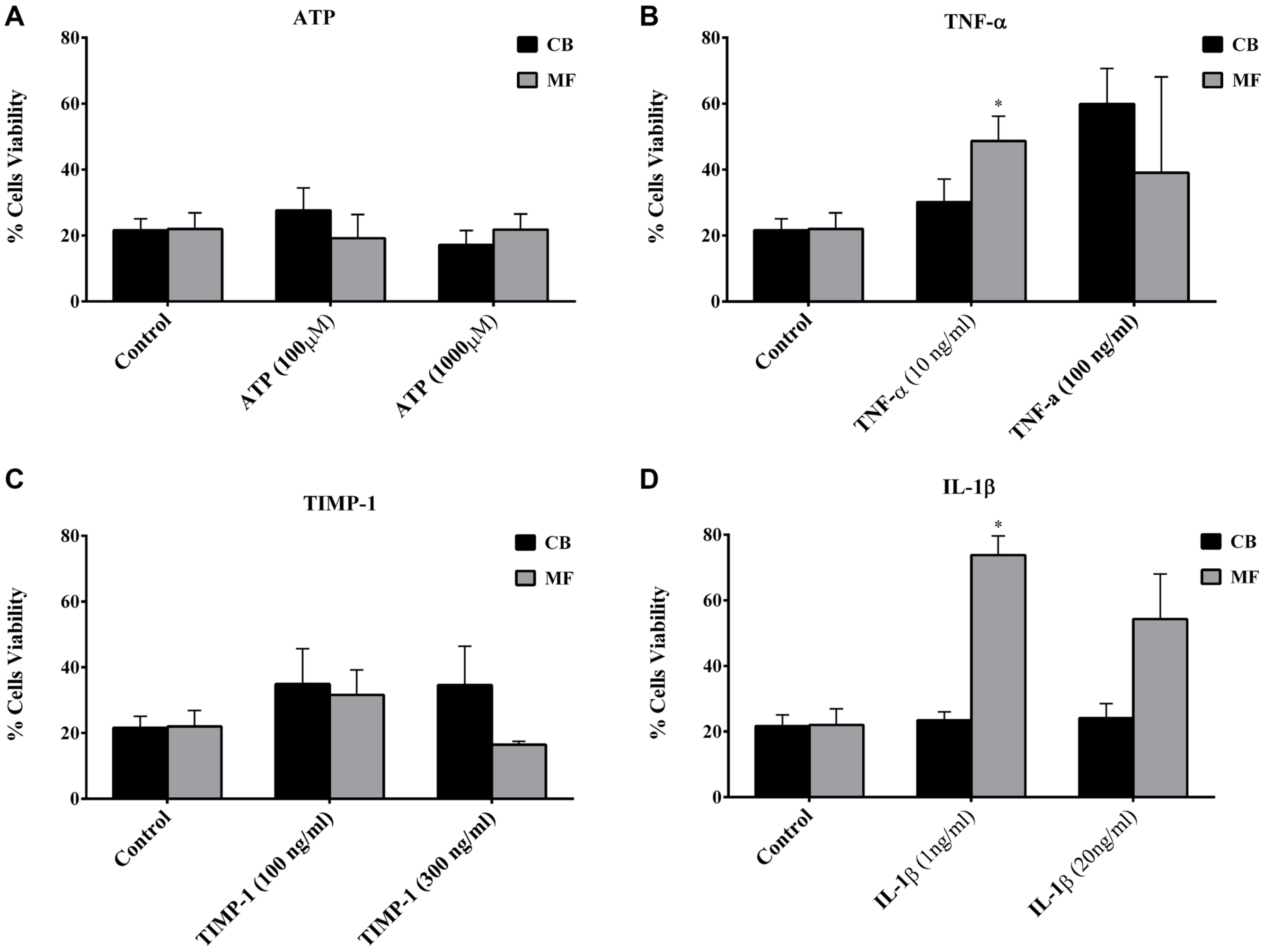

Supplementary Figure S1: Survival of MF- and CB-derived $CD34^+$ cells after *in vitro* treatment with increasing doses of inflammatory factors. $CD34^+$ cells from MF patients (n = 4) or CB (n = 3) were *in vitro* treated for 4 days with factors alone (ATP (100, 1000 μM), TNF-α (10,100 ng/mL), TIMP-1 (100,300 ng/mL) and IL-1β (1,20 ng/mL)) and the percentage of cell viability was assessed after AnnexinV/PI staining, as described in methods. (*$p \leq 0.05$ *vs untreated cells*).

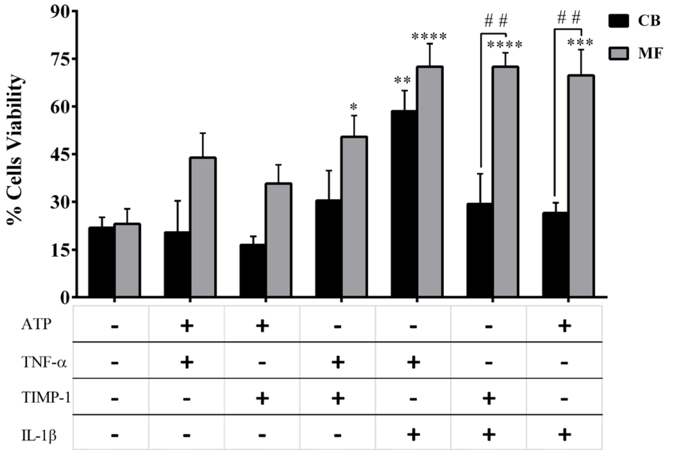

Supplementary Figure S2: Survival of CD34$^+$ cells from MF patients is increased by various combinations of pro-inflammatory factors. CD34$^+$ cells from MF patients (n = 20) or CB (n = 8) were *in vitro* treated for 4 days with factors two by two and the percentage of cell viability was assessed after AnnexinV/PI staining, as described in Methods. Cells viability of MF-derived CD34$^+$ cells was significantly increased by IL-1β + TNF-α, IL-1β + TIMP-1, TNF-α + TIMP-1 and IL-1β + ATP as compared with untreated cells. Conversely, only the IL-1β + TNF-α combination was effective in stimulating the *in vitro* survival of the CB-derived CD34$^+$ cells. Comparing MF vs CB, IL-1β + TIMP-1 and IL-1β + ATP significantly promoted the survival of the MF-derived cells. All data are presented as mean ± SEM. (**p ≤ 0.05; **p ≤ 0.01; ***p ≤ 0.001; ****p ≤ 0.0001 vs untreated cells (CTR)*) (*##p ≤ 0.01 vs CB*).

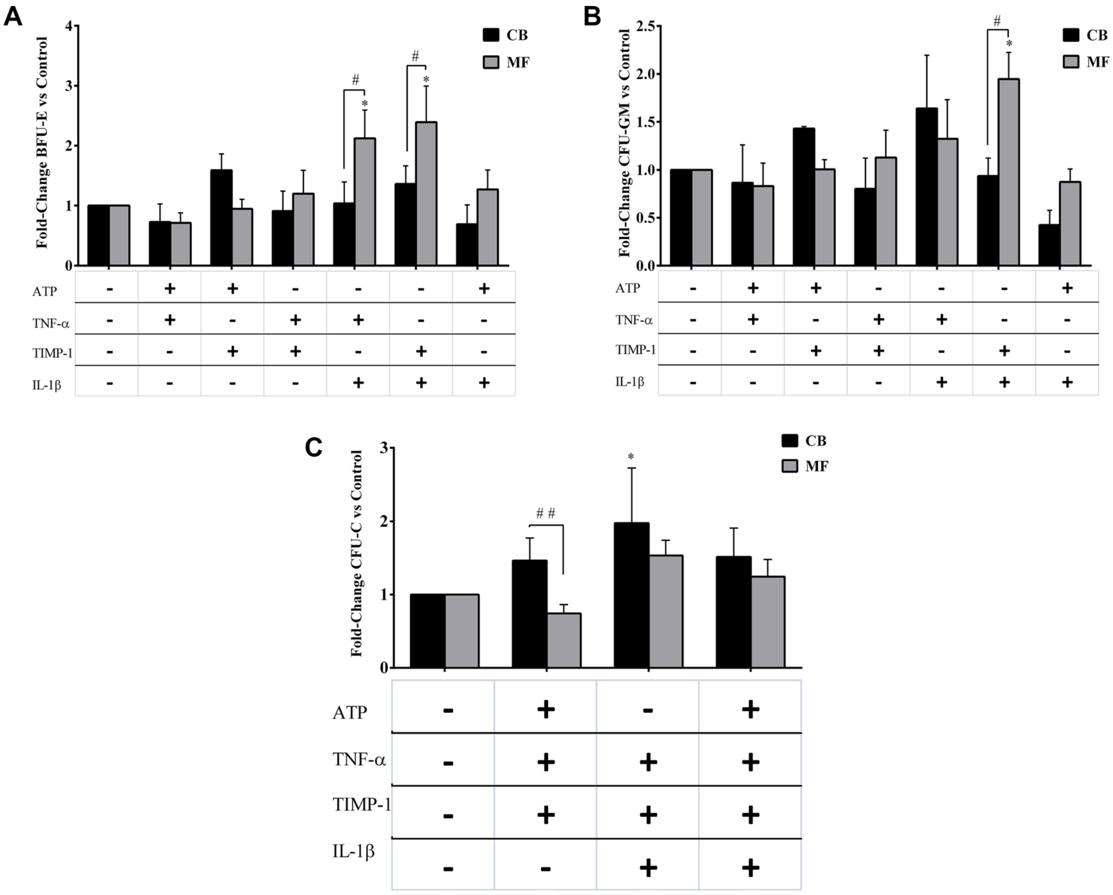

Supplementary Figure S3: Effects of inflammatory factors on BFU-E/CFU-GM growth from MF- and CB-derived CD34$^+$ cells. Circulating CD34$^+$ cells were isolated from MF patients (n = 20) and CB units (n = 8) and cultured in semisolid medium in the presence of the selected two-by-two or multiple pro-inflammatory factors. After 14 days, the BFU-E/CFU-GM (**A** and **B**) and the total CFU-C (**C**) output was assessed. (A) When IL-1β + TIMP-1 and IL-1β + TNF-α were added in culture, the BFU-E growth of MF-derived, but not CB-derived, CD34$^+$ cells was significantly increased as compared with the untreated samples and the CB counterparts. None of the others combinations were effective. (B) The growth of CFU-GM from MF-derived CD34$^+$ cells showed the same pattern displayed by BFU-E. (C) When various combinations of inflammatory factors were tested, MF-derived CD34$^+$ cells showed a decreased number of CFU-C compared to CB in response to ATP, TNFα, and TIMP. All data are presented as mean ± SEM. (**p ≤ 0.05 vs untreated cells*) (*#p ≤ 0.05; ##p ≤ 0.01 vs CB*).

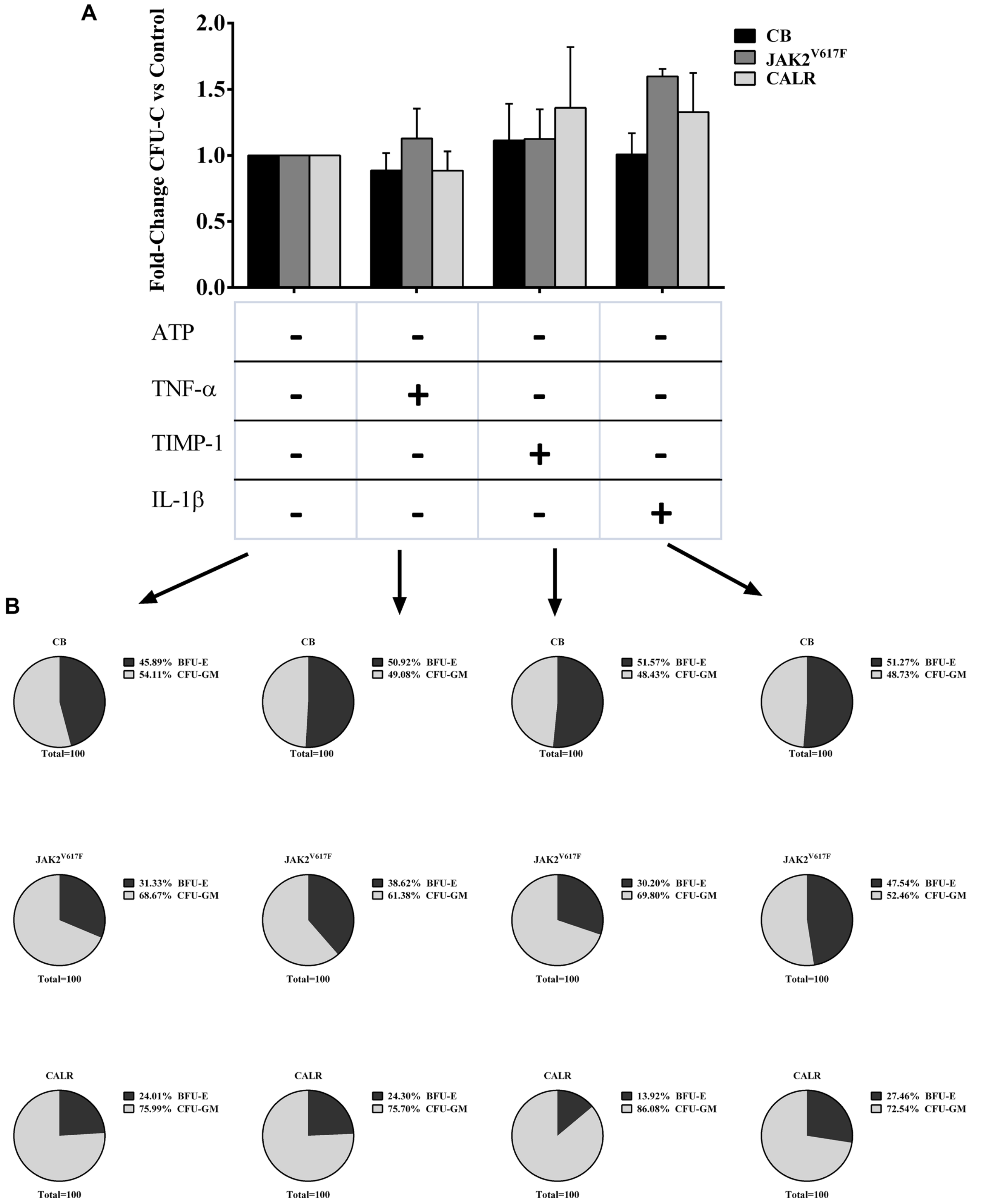

Supplementary Figure S4: Effects of pro-inflammatory factors alone on CFU-C growth of CD34$^+$ cells from *JAK2^{V617F}* and *CALR* mutated patients. (**A**) When CFU-C growth of CD34$^+$ cells from *JAK2^{V617F}* (*n* = 10) and *CALR* (*n* = 6) mutated patients was assessed in the presence of factors alone, no differences were observed between CB samples and between the two mutated groups. (**B**) Colony composition analysis demonstrated that only IL-1β enhanced the erythroid compartment of the *JAK2^{V617F}* mutated group.

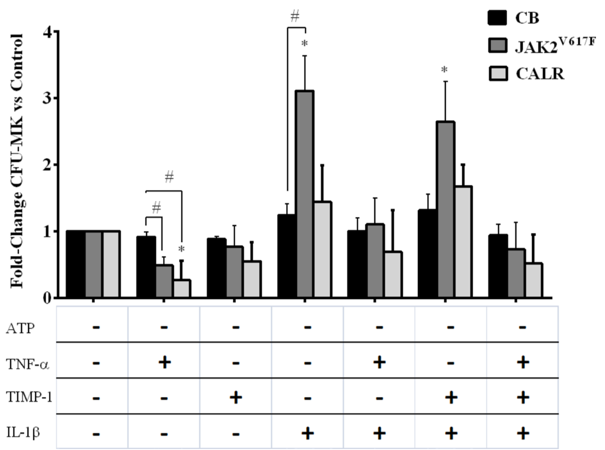

Supplementary Figure S5: CFU-MK growth according to mutation status. CFU-MK growth from *JAK2*^{V617F} (*n* = 6) and *CALR* (*n* = 4) mutated patients was significantly inhibited by TNF-α as compared with CB counterparts. By contrast, IL-1β and IL1β + TNF-α stimulated the CFU-MK growth of *JAK2*^{V617F} mutated patients (*$*p \leq 0.05$ vs untreated cells*) (*$^{\#}p \leq 0.05$; $^{\#\#}p \leq 0.01$ JAK2^{V617F}/CALR mutated patients vs CB*).

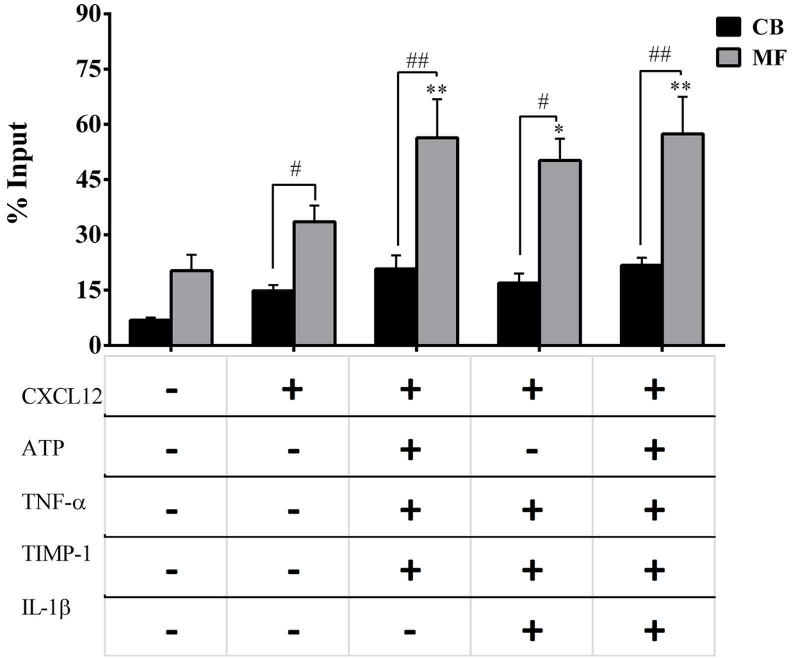

Supplementary Figure S6: Various combinations of pro-inflammatory factors significantly promote migration of MF-derived CD34⁺ cells. When migration toward multiple combinations of factors + CXCL12 was analysed, the migration ability of MF-derived (*n* = 15), but not CB-derived (*n* = 8), CD34⁺ cells was significantly increased. (*$*p \leq 0.05$; $**p \leq 0.01$ vs spontaneous migration*) (*$^{\#}p \leq 0.05$;$^{\#\#}p \leq 0.01$ vs CB*). Results are expressed as mean percentages ± SEM of input.

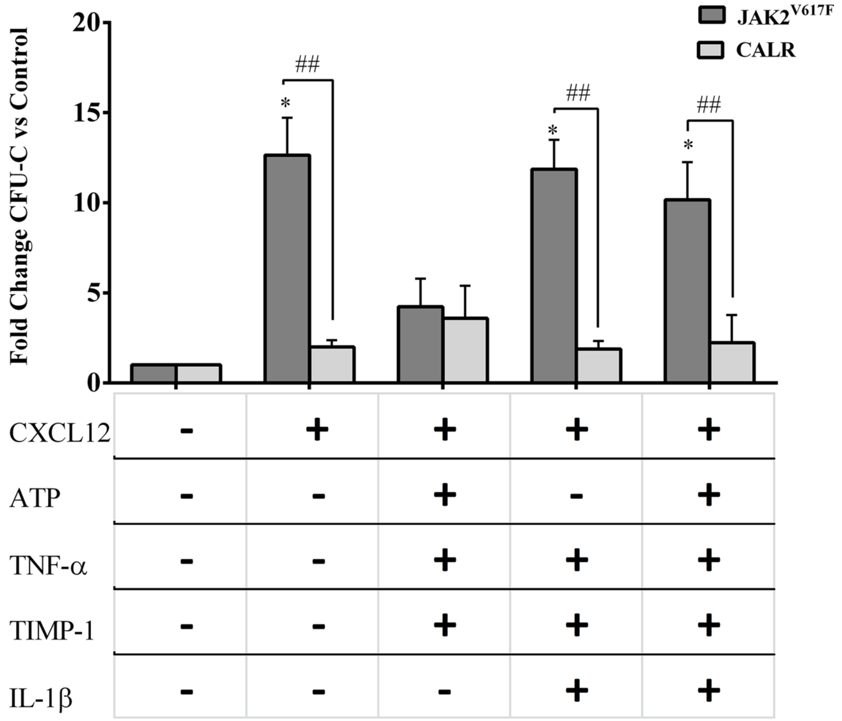

Supplementary Figure S7: CFU-C post-migration assay according to mutation status. The clonogenic potential of $CD34^+$ cells from *$JAK2^{V617F}$* (*n = 8*)/*CALR* (*n = 6*) mutated patients after migration toward CXCL12 alone or various combinations of pro-inflammatory factors + CXCL12 (CFU-C post-migration) is shown. After migration toward various combinations of pro-inflammatory factors, only the *$JAK2^{V617F}$*-derived $CD34^+$ cells show significantly increased clonogenic potential. Results are expressed as mean fold change of CFU-C ± SEM. (**$p \leq 0.05$ vs spontaneous migration*) (*$^{\#\#}p \leq 0.01$ $JAK2^{V617F}$ vs CALR mutated patients*).

Supplementary Table S1: The following MoAbs were used to phenotypically characterize the MF- and CB-derived cells

- anti-CD34 (clone 8G12)
- anti-CD38 (clone HIT2)
- anti-CD47 (clone B6H12)
- anti-CD45 (clone HI30)
- anti-CD184 (CXCR4; clone 12G5)
- anti-CD49d (clone 9F10)
- anti-CD44 (clone G44-26)
- anti-CD41a (clone HIP8)

All from BD Biosciences (San Jose, CA USA)

- anti-CD63 (TIMP-1 receptor; clone H5C6) from eBioscience (San Diego, CA USA),
- anti-CD133 (clone AC133) (Miltenyi Biotech, Bologna, Italy).

Negative controls were isotype-matched irrelevant MoAbs (BD Biosciences and Miltenyi Biotech).
